# 2,3-Oxidosqualene cyclase protects liver cells from the injury of intermittent hypoxia by regulating lipid metabolism

**DOI:** 10.1007/s11325-015-1167-1

**Published:** 2015-04-09

**Authors:** Yue-qiao Zhen, Yu-min Wu, Yan-hong Sang, Yan Wang, Qiu-yan Song, Ling Yu, Xiao-juan Rao, Rui-hong Dong

**Affiliations:** Department of Endocrinology, The Fifth Affiliated Hospital of Zhengzhou University, No. 3 Rehabilitation Street, Zhengzhou, 450052 People’s Republic of China

**Keywords:** Intermittent hypoxia, Obstructive sleep apnea, 2,3-Oxidosqualene cyclase, Triglyceride

## Abstract

**Purpose:**

2,3-Oxidosqualene cyclase (OSC), an important enzyme of cholesterol biosynthesis, catalyzes the highly selective cyclization of 2,3-monoepoxysqualene to lanosterol. Intermittent hypoxia (IH) is a hallmark feature in obstructive sleep apnea (OSA) which is increasingly recognized as an independent risk factor for liver injury. The aim of this study was to determine the effect of IH on OSC expression and evaluate the role of OSC in the IH-induced apoptosis in hepatic cell line human liver cell (HL-02).

**Methods:**

HL-02 cells were exposed to normoxia or IH. Cell Counting Kit-8 (CCK-8) assay was used to value cell proliferation, and flow cytometry was used to determine cell apoptosis. The expression of OSC messenger RNA (mRNA) was evaluated by quantitative real-time PCR, and the expression of OSC protein was determined by Western blot. To further investigate the function of OSC in IH-induced apoptosis, oxidosqualene cyclase-enhanced green fluorescence protein (OSC-EGFP) plasmid was constructed to over-express OSC protein. Triglyceride content in HL-02 cells was analyzed by oil red staining or Triglyceride Quantification Kit.

**Results:**

We found that IH inhibited HL-02 cell proliferation and accelerated cell apoptosis. IH decreased OSC expression, and over-expression of OSC could protect HL-02 cells against the IH-induced hepatic cell injury. Moreover, over-expression of OSC could attenuate IH-induced cellular triglyceride accumulation.

**Conclusions:**

These findings suggest that OSC are involved in IH-induced hepatic cell injury. These results may contribute to the further understanding of the mechanism underlying the liver injury in OSA patients.

## Introduction

Obstructive sleep apnea (OSA) is caused by recurrent closure of the upper airway during sleep, which leads to repetitive intermittent hypoxia (IH) [1]. OSA is a common disorder, present in 2 % of women and 4 % of men in the population [2, 3]. Obesity is a major risk factor for OSA, and prevalence of OSA exceeds 50 % in obese men [4, 5]. OSA has been associated with an increased risk of hypertension, type 2 diabetes, dyslipidemia, and atherosclerosis, independent of underlying obesity [6–9]. Moreover, accumulating evidence showed that OSA is associated with elevation in liver enzymes and biopsy evidence of liver injury. The severity of liver injury in patients with OSA directly correlates with the severity of the hypoxic insult [10–13].

Although OSA has been proposed to be a potential risk factor of liver injury, the underlying mechanism is still unobvious. In the study conducted by Li J et al., after the leptin-deficient obese (ob/ob) mice were exposed to IH during the light phase (9 a.m.–9 p.m.) for 12 weeks, IH caused a 30 % increase in liver triglyceride and phospholipid, whereas liver cholesterol content was unchanged. Gene expression analysis showed that IH up-regulated sterol regulatory element-binding protein (SREBP)-1, a master regulator of lipogenesis. Expression of major genes of cholesterol biosynthesis, SREBP-2 and 3-hydroxy-3-methylglutaryl-CoA reductase, was unchanged [14, 15]. In this study, we evaluated the impact of IH on the expression of 2,3-oxidosqualene cyclase (OSC), another important enzyme of cholesterol biosynthesis, in the human hepatic cell line HL-02. Furthermore, we analyzed the role of OSC in the hepatic injury caused by IH.

OSC is downstream of cholesterol biosynthesis. OSC catalyzes the highly selective cyclization of 2,3-monoepoxysqualene (MOS) to lanosterol, the first sterol to be formed. OSC also catalyzes cyclization of 2,3;22,23-diepoxysqualene (DOS), which itself is derived from MOS, to 24(S),25-epoxylanosterol, the immediate precursor of 24(S),25-epoxycholesterol. Synthesis of 24(S),25-epoxycholesterol is favored over cholesterol synthesis under conditions of partial OSC inhibition [16–18]. Although many studies revealed IH caused dyslipidemia, the impact of IH on the expression of OSC and its role in the IH-induced hepatic injury has not been reported. In the present study, we investigated the effect of IH on the proliferation of HL-02 cells and OSC expression. Moreover, we analyzed the role of OSC in the IH-induced hepatic injury.

## Materials and methods

### Cell culture and intermittent hypoxia treatment

The HL-02 cells were obtained from the Institute of Biochemistry and Cell Biology. The cells were cultured in Dulbecco’s modified Eagle’s medium (DMEM, Gibco) containing 20 % fetal bovine serum at 37 °C in 5 % CO_2_ incubator. IH exposure was conducted using a custom-designed computer controlled incubator chamber connected to a BioSpherix OxyCycler (BioSpherix, Redfield, NY, USA). Cells were maintained in the hypoxic chamber in which O_2_ levels were alternated between 2 % for 5 min and 21 % for 10 min. Cells in the control group were maintained in normoxic conditions (21 % O_2_ and 5 % CO_2_). In order to analyze the effect of oxygen concentrations on the OSC expression, cells were maintained in the cycle of 0 % O_2_ for 5 min and 21 % O_2_ for 10 min, cycle of 2 % O_2_ for 5 min and 21 % O_2_ for 10 min, cycle of 5 % O_2_ for 5 min and 21 % O_2_ for 10 min, and cycle of 10 % O_2_ for 5 min and 21 % O_2_ for 10 min or normoxia, respectively.

### Cell growth assay

Cell Counting Kit-8 (CCK-8) assays were used to determine cell proliferation according to the manufacturer’s instructions. 1 × 10^4^ HL-02 cells were seeded in each pool of 96-well plates. The cells were exposed to IH or normoxia for different time intervals. After cultivation, CCK-8 reagents (WST-8) were added to each well at different time points and then incubated for 1.5 h at 37 °C in the absence of light. Then the WST-8 was reduced to yellow formazan by the dehydrogenase in the mitochondrial of living cells. The absorbance of dissolved yellow formazan was measured at a test wavelength of 450 nm and a reference wavelength of 630 nm with a microplate reader (Bio-Rad).

### Cell apoptosis assay

HL-02 cells were plated in six-well plates and incubated for 24 h. Then the cells were exposed to IH or normoxia for 4 days. After IH treatment, cells were harvested by trypsinization and washed twice in phosphate buffer saline (PBS). When the cells undergo apoptosis, the phosphatidylserine inverts from the inner membrane surface to the outer membrane surface and binds with fluorescein isothiocyanate (FITC)-labeled annexin V. After staining with the combination of annexin V/FITC and propidium iodide (PI) (annexin V-FITC apoptosis detection kit, BD Pharmingen), the cells were immediately analyzed by flow cytometry (FACSCalibur, Becton Dickinson).

### Real-time PCR

Total ribonucleic acid (RNA) was extracted from treated HL-02 cells using Trizol reagent according to the manufacturer’s instructions. The complementary deoxyribonucleic acid (cDNA) was synthesized from total RNA as template using a high-capacity cDNA reverse transcription kit (Takara, Japan). Real-time PCR was performed using Fast SYBR^®^ Green Master Mix (Applied Biosystems). The PCR primers for OSC were as follows: 5′-TGCAGAATCAGTGTCCGTCC-3′, 5′-TAGGTATGCCCCA TGCAAGC-3′. The messenger RNA (mRNA) signal was normalized to β-actin signal.

### Western blot analysis

After IH treatment, cells were lysed with a solution containing Tris-HCl (50 mmol/L, pH 6.8), sodium dodecyl sulfonate (SDS) (2 % *w*/*v*), glycerol (10 %), and dithiothreitol (10 mmol/L), supplemented with protease inhibitor mix (Thermo Fisher). Cell lysates were centrifuged at 12,000×*g* for 30 min. Equal amounts of the protein (50 μg) were resolved by sodium dodecyl sulfonate-polyacrylamide gel electrophoresis (SDS-PAGE) and transferred onto nitrocellulose membranes. The membranes were blocked with 5 % non-fat dry milk in Tris buffered saline (TBS) for 1 h at room temperature and then incubated with primary antibodies against OSC, hypoxia-inducible factor-1 (HIF-1), SREBP-1, fatty acid synthase (FAS), or β-actin at 4 °C overnight. Membranes were washed and treated with appropriate secondary antibodies for 1 h at room temperature. The immunocomplexes were detected with the enhanced chemiluminescence plus kit.

### Plasmid constructs

The OSC cDNA clone was cloned from the cDNA of HepG2 cell line, performed by polymerase chain reaction (PCR) then inserted coding sequence in the pEGFP-N1 plasmid (Invitrogen). The EGFP tag sequence was fused to the C-terminus of the proteins to facilitate further detection via confocal microscope. The plasmids were obtained using the Plasmid Maxiprep kit (Vigorous) and verified by DNA sequencing.

### Oil red staining

The HL-02 cells were cultivated on glass cover slides. After cultivation for 24 h, cells reached 90 % confluence and transfection was performed. The cells were transfected with plasmids pEGFP-N1 or oxidosqualene cyclase-enhanced green fluorescence protein (OSC-EGFP) using Lipofectamine 2000 (Invitrogen) following the instructions. After transfection for 4 h, the cells were moved to a fresh high glucose DMEM medium to remove the transfection reagent. After 24 h cultivation, the transfected cells were exposed to IH or normoxia for 4 days. After treatment, cells were fixed by 4 % paraformaldehyde for 40 min followed by washing with PBS for three times, then stained with oil red. The nuclei were counterstained with DAPI. The mounted cells were visualized using a Carl Zeiss LSM 710 confocal microscope.

### TG level assay

The HL-02 cells were seeded in six-well plates and transfected with pEGFP-N1 or OSC-EGFP. After 24 h transfection, cells were exposed to IH or normoxia for 4 days. After treatment, cells were harvested. Lipids were assayed by a Triglyceride Quantification Kit from Cayman Chemical according to the manufacturer’s instructions. The total protein level was used as normalization.

### Statistical analysis

In our study, all of the experiments were conducted three times with consistent results. The data of representative experiments are presented. In the bar figures, a mean value and standard error of multiple data points or samples were used to represent the final result. Student’s *t* test or one-way ANOVA was used in statistical analysis of the data with significance *p* < 0.05.

## Results

### IH inhibited cell proliferation and accelerated cell apoptosis

**Fig. 1:**
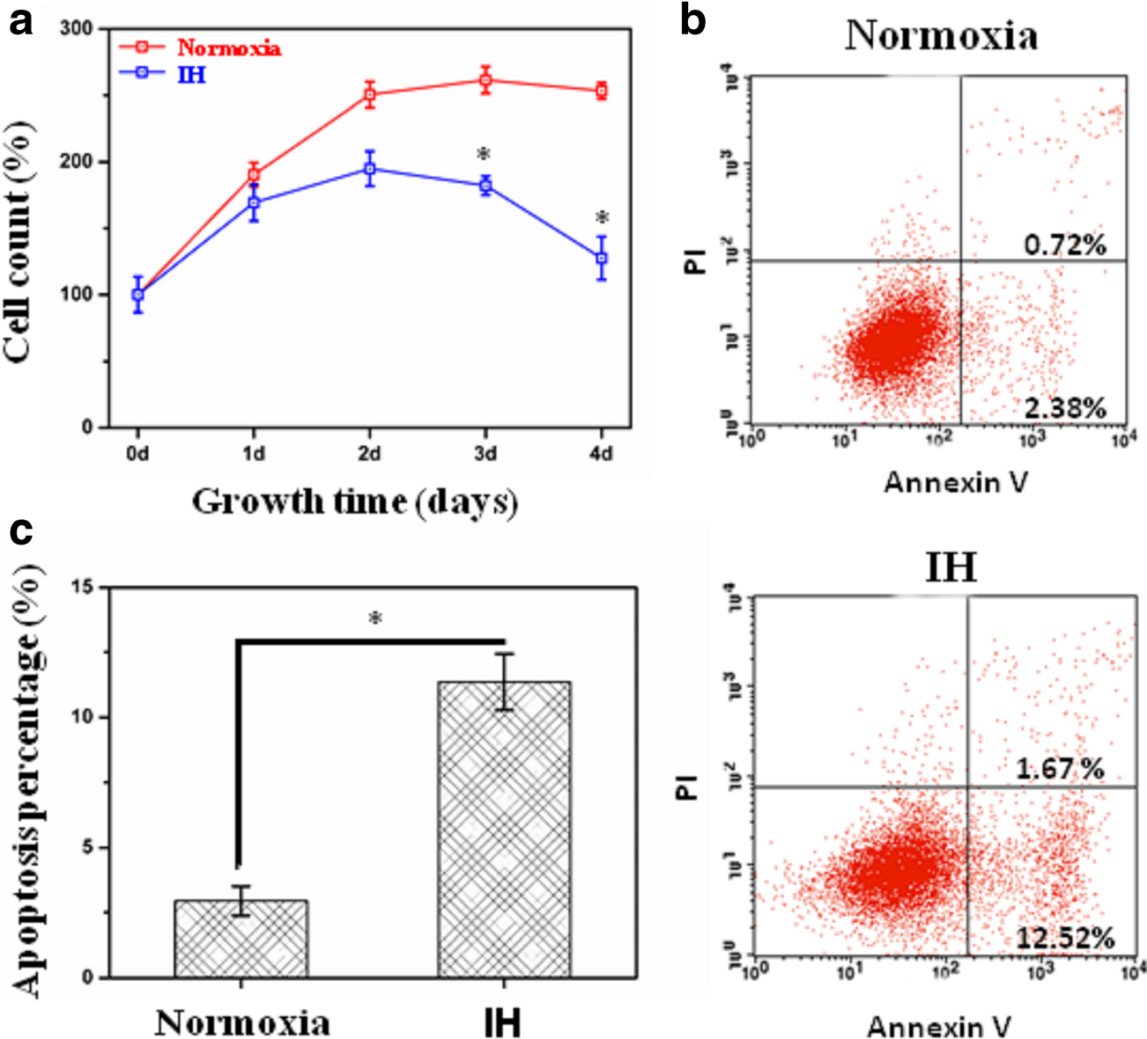
Intermittent hypoxia (IH) was toxic for HL-02 cells and inhibited their proliferation. HL-02 cells were exposed to IH (cycle of 5 min 2 % O_2_ and 10 min 21 % O_2_) or normoxia (21 % O_2_) for 4 days. **a** Effect of the IH on HL-02 cell viability. A total of 10^4^ cells were seeded in each pool of 96-well plates, and then cell viability was assayed using CCK-8 after treatment. **b** Representative flow cytometry profiles of cell apoptosis probed by annexin V binding (*horizontal*) and PI exclusion (*vertical*). **c** Percentages of apoptotic HL-02 cells after IH treatment. *p < 0.05 vs the cells grown under normoxic conditions

As compared with exposure to normoxia, HL-02 cells treated with IH had reduced cell proliferation as assessed by the CCK-8 assay (Fig. 1a). As compared with exposure to normoxia, HL-02 cells treated with IH for 4 days had increased proportions of apoptotic cells (Fig. 1b, c).

### IH decreased OSC expression in a dose-dependent manner

**Fig. 2:**
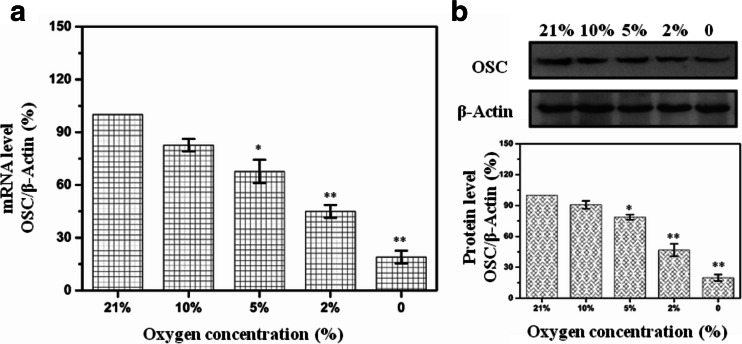
Expression of OSC in HL-02 cells treated with IH (cycles of 0, 2, 5, or 10 % O_2_ 5 min and 21 % O_2_ 10 min) for 4 days. **a** mRNA levels of OSC expression in HL-02 cells were determined with real-time quantitative PCR under different oxygen concentrations. **b** Protein levels of OSC expression in HL-02 cells were determined by Western blot after IH treatment. *p < 0.05, **p < 0.01 vs the cells grown under normoxic conditions

As shown in Fig. 2a, the mRNA level of OSC was significantly decreased in HL-02 cells treated with IH at different oxygen concentrations for 4 days. Western analysis also showed that protein level of OSC was decreased in HL-02 cells treated with IH at different oxygen concentrations for 4 days (Fig. 2b).

### IH decreased OSC expression in a time-dependent manner

**Fig. 3:**
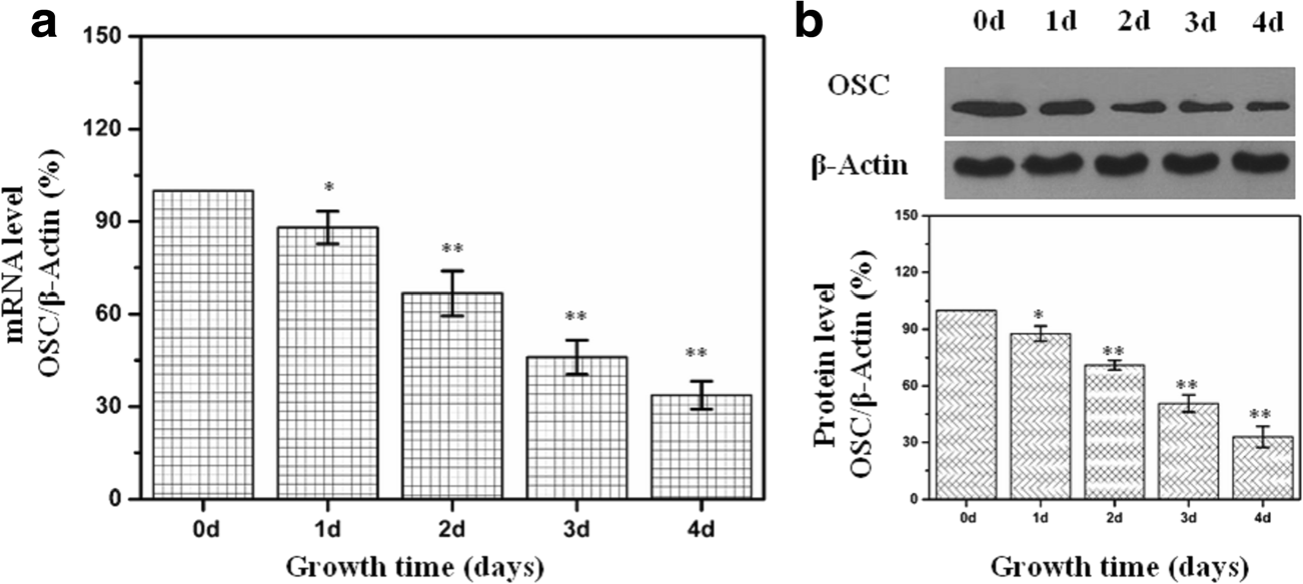
Expression of OSC in HL-02 cells treated with IH (cycle of 2 % O_2_ 5 min and 21 % O_2_ 10 min) for different times. **a** mRNA levels of OSC expression in HL-02 cells were determined with real-time quantitative RT-PCR in an IH treatment time course study. **b** Protein levels of OSC expression in HL-02 cells were determined by Western blot after IH treatment. *p < 0.05, **p < 0.01 vs the cells grown under normoxic conditions

As compared with exposure to normoxia, HL-02 cells treated with IH resulted in a decrease of OSC mRNA in a time-dependent manner (Fig. 3a). Western analysis also showed that the protein level of OSC was decreased in HL-02 cells treated with IH at different time points (Fig. 3b).

### Over-expression of OSC enhanced proliferation and decreased apoptosis in HL-02 cells treated with IH

**Fig. 4:**
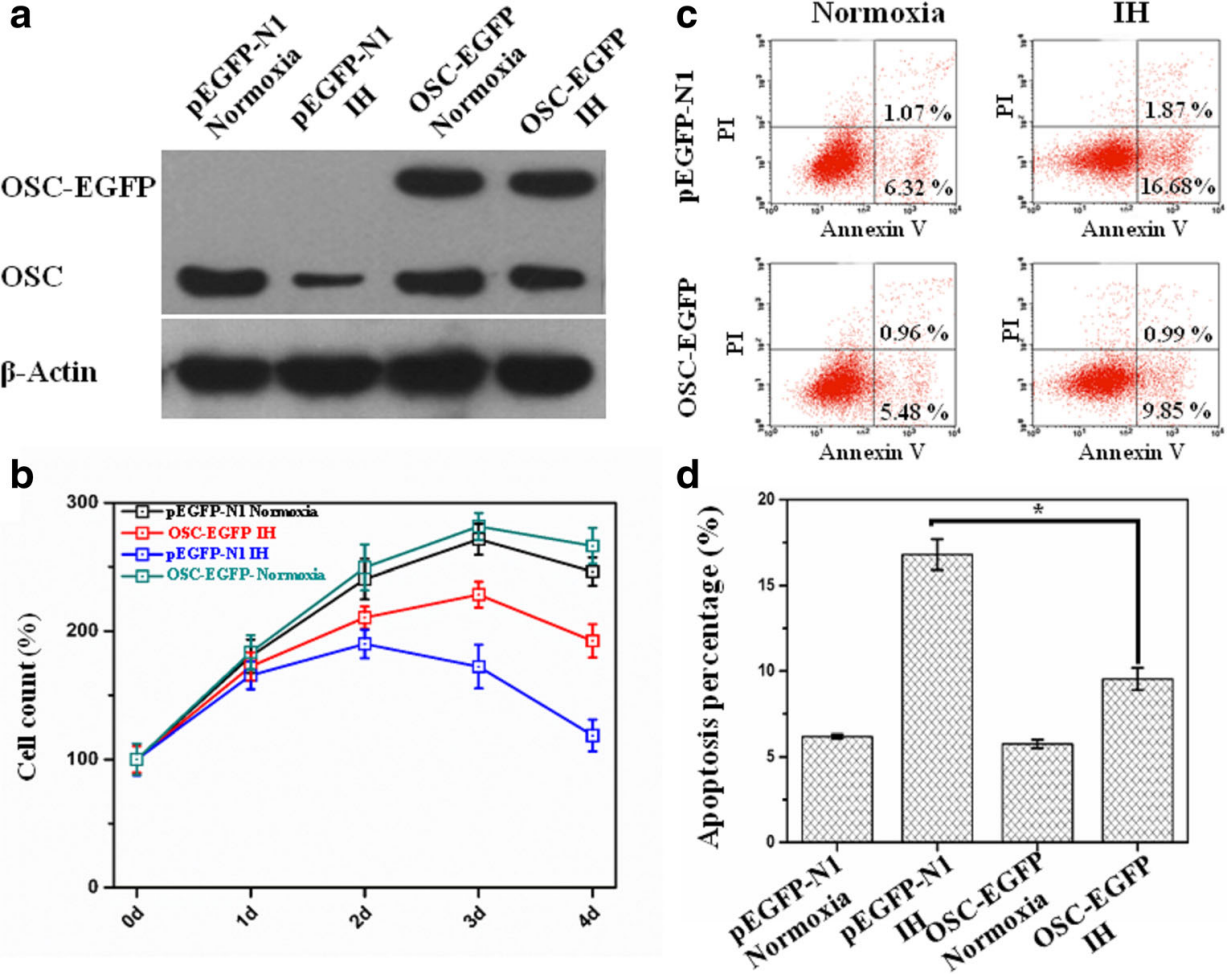
Over-expression of OSC can protect HL-02 cells from the injury of IH. After transfection, HL-02 cells were exposed to IH (cycle of 2 % O_2_ 5 min and 21 % O_2_ 10 min) or normoxia (21 % O_2_) for 4 days. **a** Protein levels of OSC in HL-02 cells transfected with pEGFP-N1 or OSC-pEGFP were determined by Western blot after IH treatment. **b** Effect of the IH on the cell viability in HL-02 cells transfected with pEGFP-N1 or OSC-PGFP. **c** Representative flow cytometry profiles of cell apoptosis probed by annexin V binding (*horizontal*) and PI exclusion (*vertical*) in HL-02 cells transfected with pEGFP-N1 or OSC-EGFP after IH treatment. **d** Percentages of apoptotic HL-02 cells transfected with pEGFP-N1 or OSC-EGFP after IH treatment. *p < 0.05 vs the cells transfected with pEGFP-N1 control plasmid

As shown in Fig. 4a, the cells transfected with OSC-EGFP had enhanced expression of OSC compared with the cells transfected with pEGFP-N1 control plasmid. OSC protein increased about twofold in the cells transfected with OSC-EGFP as compared with that in the cells transfected with pEGFP-N1.

The result of CCK-8 assay showed that the over-expression of OSC attenuated IH-induced proliferation inhibition in HL-02 cells (Fig. 4b). Apoptosis analysis also showed that over-expression of OSC resulted in the decrease of apoptotic cells as compared with that in the pEGFP-N1-transfected HL-02 cells treated with IH (Fig. 4c, d).

### Over-expression of OSC decreased TG content in HL-02 cells treated with IH

**Fig. 5:**
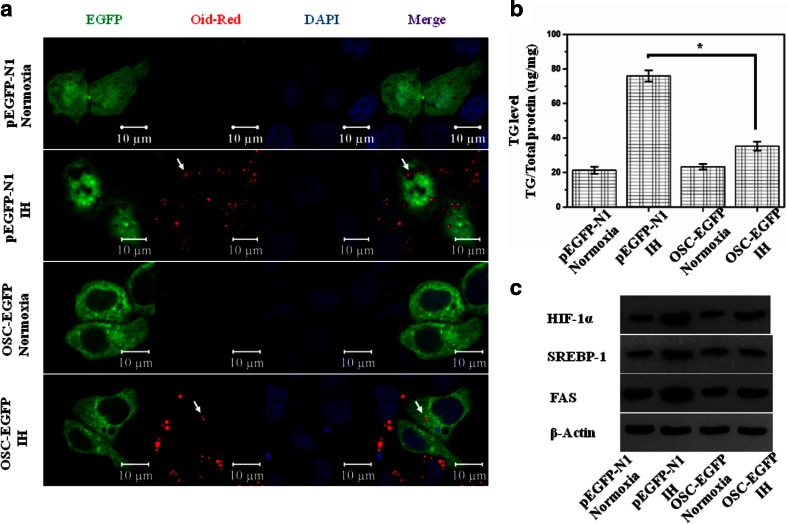
Over-expression of OSC can prevent superabundance of the lipid droplet accumulation and TG content in HL-02 cells treated with IH (cycle of 2 % O_2_ 5 min and 21 % O_2_ 10 min) or normoxia (21 % O_2_) for 4 days. **a** State of lipid droplet accumulation in HL-02 cells transfected with pEGFP-N1 or OSC-EGFP after IH treatment. **b** TG level in HL-02 cells transfected with pEGFP-N1 or OSC-EGFP after IH treatment. **c** The expression levels of HIF-1, SREBP-1, and FAS in HL-02 cells transfected with pEGFP-N1 or OSC-EGFP after IH treatment. *p < 0.05 vs the cells transfected with pEGFP-N1 control plasmid

As compared with normoxia, Triglyceride (TG) content was significantly increased after exposure to IH for 4 days in HL-02 cells transfected with pEGFP-N1 control plasmid. Over-expression of OSC did not influence the TG content in HL-02 cells under normoxic condition. However, over-expression of OSC significantly decreased the TG content in HL-02 cells treated with IH for 4 days (Fig. 5a, b). Moreover, we evaluated the expression of HIF-1, SREBP-1, and FAS by Western blot. We found that IH increased the expression of HIF-1, SREBP-1, and FAS. Over-expression of OSC attenuated the IH-induced increase in the expression of HIF-1, SREBP-1, and FAS (Fig. 5c).

## Discussion

Clinical literature suggests that there is an association between OSA and hepatic cell injury, and the severity is directly proportional to the hypoxic stress of OSA [19–21]. Polotsky et al. studied 90 consecutive patients who suffered from OSA and reported that nocturnal oxygen desaturation might predispose to hepatic inflammation, hepatocyte ballooning, and liver fibrosis [22]. The association between OSA and hepatic cell injury has been further supported by animal experiments. The findings in the present study extend these observations by demonstrating that exposure of hepatic cells HL-02 to IH led to a marked cell growth inhibition and elevation in cell apoptosis.

Many studies have shown that IH results in hyperlipidemia and IH-induced hyperlipidemia contribute to liver injury. IH was associated with increased serum total cholesterol and triglycerides. Similarly, an increase in liver lipid content was noted [23–25]. Therefore, the expression profile of the genes involved in lipid metabolism was explored under IH conditions. Studies showed that IH induces HIF-1 in the liver which, in turn, activates SREBP-1. In concert, SREBP-1 induces gene expression of SCD-1, independent of SREBP-2, to enhance triglyceride and phospholipid biosynthesis [26]. In this study, we evaluated the impact of hypoxia on the expression of OSC, an important enzyme of cholesterol biosynthesis, in the human hepatic cell line HL-02. We demonstrated for the first time that OSC mRNA, as well as protein, was decreased under intermittent hypoxic conditions. In order to investigate the role of OSC in the IH-induced apoptosis, OSC was over-expressed in the HL-02 cells. We found that over-expression of OSC could protect the IH-induced hepatic cell injury. To further analyze the mechanism through which over-expression of OSC protect the HL-02 cells against IH-induced apoptosis, the lipid content in the cells was determined. The results showed that IH could induce the lipid droplet accumulation and TG content, and over-expression of OSC can prevent superabundance of the lipid droplet accumulation and TG content in IH-treated HL-02 cells.

OSC catalyzes the conversion of the 2,3-oxidosqualene into lanosterol in mammals [27]. OSC occupies a unique position in the cholesterol biosynthetic pathway. In fact, it catalyzes not only the conversion of 2,3-monoepoxysqualene to lanosterol but also the cyclization of 2,3;22,23-diepoxysqualene to 24(S),25-epoxylanosterol which is subsequently transformed into 24(S),25-epoxycholesterol [28]. The synthesis of 24(S),25-epoxycholesterol is favored over cholesterol synthesis under condition of partial OSC inhibition. 24(S),25-epoxycholesterol is a potent activator of the liver X receptor (LXR). LXR activation is known to increase transcription of a number of genes important in the regulation of hepatic lipid metabolism, including SREBP-1 and fatty acid synthase [29]. In our study, we found that IH increased the expression of HIF-1, SREBP-1, and FAS. OSC could inhibit the IH-induced expression of HIF-1, SREBP-1, and FAS. Therefore, IH-mediated inhibition of OSC may increase the synthesis of 24(S),25-epoxycholesterol and result in the synthesis and accumulation of hepatic triglycerides through HIF-1, SREBP-1, and FAS activation. Increased accumulation of triglycerides predisposes HL-02 cells to apoptosis. In addition, a recent study reports that OSC protected self-renewing T2EC cells from apoptosis and blocked their differentiation program. This study indicated that OSC itself or the product of this enzyme may play a part in apoptosis process [30].

In conclusion, the expression of OSC was inhibited under hypoxic conditions. Over-expression of OSC could protect HL-02 cells against hypoxia-induced apoptosis. The mechanism underlying the protective effect of OSC on the IH-induced apoptosis may be that over-expression of OSC could inhibit the accumulation of TG in the HL-02 cells.
